# The Combat Deepens

**Published:** 1883-06

**Authors:** 


### THE COMBAT DEEPENS.

The New York codical war deepens in intensity. The roar of artillery is beginning to wake the welkin, and the end is not yet. New York should have contended for her rights under the old flag, and all would have been well. Alas! the spirit and wisdom of Little Aleck was not there. We sincerely sympathize with them in their troubles, and it is in the order of a handsome thing for their brethren of the South to pray earnestly that they be delivered from the horrors of reconstruction and an inundation of infamous carpet-baggers.

